# Characterization of Spent Coffee Grounds’ Polyphenol Fraction and Its Potential as a Low-Cost Tool for Bioactivity-Guided Nephroprotective Modulation of Gut–Kidney Axis and NF-κB/Nrf2/TGF-β Signaling in Hypertension-Associated Kidney Injury

**DOI:** 10.3390/antiox15070889

**Published:** 2026-07-17

**Authors:** Fahrul Nurkolis, Dante Saksono Harbuwono, Yulia Wardhani, Edwin Hadinata, Siti Nur Rohmah, Metalia Puspitasari, Lucia De Luca, Raffaele Romano, Danny Pratama Kuswadi, Raymond Rubianto Tjandrawinata, Eka Ginanjar, Pringgodigdo Nugroho, Antonello Santini

**Affiliations:** 1 Faculty of Medicine, Universitas Airlangga, Surabaya 60131, Indonesia; 2 Medical Research Center of Indonesia, Surabaya 60281, Indonesia; 3 Institute for Research and Community Service, State Islamic University of Sunan Kalijaga (UIN Sunan Kalijaga), Yogyakarta 55281, Indonesia; 4 Fahrul Institute for Innovation and Research (FIIR), Yogyakarta 55281, Indonesia; 5 Division of Endocrinology, Metabolism, and Diabetes, Department of Internal Medicine, Faculty of Medicine, Universitas Indonesia, Dr. Cipto Mangunkusumo National Referral Hospital, Jakarta 10430, Indonesia; 6 Metabolic, Cardiovascular, and Aging Cluster, Faculty of Medicine Universitas Indonesia, The Indonesian Medical Education and Research Institute, Jakarta 10430, Indonesia; 7 Nephrology and Hypertension Division, Department of Internal Medicine, Faculty of Medicine, Public Health and Nursing Gadjah Mada University/Dr. Sardjito General Hospital, Yogyakarta 55281, Indonesia; 8 School of Medicine, Faculty of Medicine, Universitas Ciputra Surabaya, Surabaya 60219, Indonesia; 9 Department of Agricultural Sciences, University of Naples Federico II, Piazza Carlo di Borbone I, 80055 Portici, Italy; 10 School of Bioscience, Technology and Innovation, Atma Jaya Catholic University of Indonesia, Jakarta 12930, Indonesia; 11 Division of Cardiology, Department of Internal Medicine, Faculty of Medicine, Universitas Indonesia, Jakarta 10430, Indonesia; 12 Division of Nephrology and Hypertension, Department of Internal Medicine, Faculty of Medicine Universitas Indonesia/Dr. Cipto Mangunkusumo National General Hospital, Jakarta 10430, Indonesia; 13 Department of Pharmacy, University of Napoli Federico II, Via Domenico Montesano, 49, 80131 Napoli, Italy

**Keywords:** chlorogenic acid derivatives, caffeoylquinic acids, bioactivity-guided fractionation, gut–kidney axis, NF-κB/Nrf2/TGF-β signaling, hypertensive kidney injury

## Abstract

Background: Hypertension-associated kidney injury is driven by oxidative stress, inflammation, fibrosis, and gut-derived uremic toxins via the gut–kidney axis. Spent coffee grounds (SCG) represent a sustainable source of bioactive polyphenols with potential nephroprotective effects. This study aimed to characterize a bioactivity-guided SCG polyphenol fraction and evaluate its therapeutic potential. Methods: Bioactivity-guided fractionation was performed to enrich active polyphenols, followed by in silico target prediction and in vivo validation using L-NAME-induced hypertensive rats treated for 8 weeks. Renal function, oxidative stress, inflammatory and fibrotic markers, gut-derived metabolites, and endothelial function were assessed. Results: The enriched fraction was dominated by chlorogenic acid derivatives and showed predicted interactions with NF-κB, Keap1/Nrf2, TGF-β receptor, ACE, and AT1 receptor. In hypertensive rats, treatment significantly lowered blood pressure, improved renal function, reduced oxidative stress, inflammation, fibrosis, and gut-derived uremic toxins, while restoring Nrf2 activity, short-chain fatty acid production, and endothelial function. Across multiple biological endpoints, the polyphenol-enriched fraction consistently demonstrated greater efficacy than the crude extract. Conclusions: SCG polyphenol fraction shows strong nephroprotective potential via gut–kidney axis modulation and NF-κB/Nrf2/TGF-β regulation, supporting its development as a low-cost therapeutic candidate.

## 1. Introduction

Hypertension-associated kidney injury represents a major global health burden and is a leading contributor to the progression of chronic kidney disease (CKD) and end-stage renal failure [1,2]. The pathophysiology of hypertensive nephropathy is multifactorial, involving persistent hemodynamic stress, endothelial dysfunction, oxidative damage, chronic inflammation, and progressive fibrosis [3,4]. At the molecular level, dysregulation of key signaling pathways, particularly nuclear factor kappa B (NF-κB), nuclear factor erythroid 2-related factor 2 (Nrf2), and transforming growth factor-beta (TGF-β), plays a central role in driving renal injury [5]. NF-κB activation promotes pro-inflammatory cytokine release, Nrf2 impairment weakens antioxidant defenses, and TGF-β signaling accelerates extracellular matrix deposition and fibrosis, collectively leading to irreversible renal damage.

In recent years, the gut–kidney axis has emerged as an increasingly recognized but still underexplored contributor to hypertension-induced renal dysfunction. Alterations in gut microbiota composition (dysbiosis) promote the accumulation of gut-derived uremic toxins such as indoxyl sulfate, which exacerbate oxidative stress, inflammation, and endothelial dysfunction [6]. Conversely, beneficial microbial metabolites, including short-chain fatty acids (SCFAs), exert protective effects by modulating immune responses, improving vascular function, and enhancing metabolic homeostasis [7,8]. This bidirectional interplay highlights the importance of targeting both systemic and gut-mediated mechanisms in developing effective nephroprotective strategies.

Despite advances in pharmacological therapy, effective management of hypertension-associated kidney injury remains challenging because current treatments do not fully prevent disease progression and may be associated with adverse effects or high costs. Consequently, increasing attention has been directed toward sustainable plant-derived polyphenols with pleiotropic biological activities [9,10]. Among these, spent coffee grounds (SCG), an abundant agro-industrial by-product rich in chlorogenic acids and related phenolics, have emerged as a promising source of bioactive compounds. Although previous studies have demonstrated antioxidant and metabolic benefits of SCG extracts [11,12,13], most investigations have relied on crude extracts with variable chemical composition, limiting reproducibility, mechanistic understanding, and translational potential.

Several important knowledge gaps therefore remain. First, bioactivity-guided fractionation has rarely been applied to enrich the pharmacologically active polyphenols present in spent coffee grounds. Second, few studies have established clear links between the chemical composition of enriched fractions and their biological effects in hypertension-associated kidney injury. Third, the influence of spent coffee ground-derived polyphenols on the coordinated regulation of the gut–kidney axis together with the NF-κB/Nrf2/TGF-β signaling network remains poorly understood.

Accordingly, the aim of this study was to evaluate the nephroprotective effects of a bioactivity-guided polyphenol-enriched fraction derived from spent coffee grounds in an L-NAME-induced model of hypertension-associated kidney injury and to investigate the molecular and metabolic mechanisms underlying these effects. We hypothesized that enrichment of chlorogenic acid-rich polyphenols would produce greater nephroprotective effects than the corresponding crude extract by simultaneously attenuating oxidative stress, inflammation, and fibrosis while improving gut–kidney axis homeostasis. To test this hypothesis, the enriched fraction was chemically characterized and subsequently evaluated using complementary computational and experimental approaches.

## 2. Materials and Methods

### 2.1. Materials and Chemicals

SCG were collected from a single local coffee processing facility in Yogyakarta, Indonesia, that routinely prepares 100% Robusta (*Coffea canephora* Pierre ex A. Froehner) coffee using a standardized espresso extraction protocol (approximately 18 g of ground coffee extracted to 36 mL beverage under ~9 bar pressure). The coffee beans were roasted to a medium roast prior to brewing. The collected SCG were subsequently air-dried at room temperature (25 ± 2 °C) to constant weight and finely ground using a laboratory mill (Retsch GmbH, Haan, Germany) to obtain a uniform particle size (<500 µm). To ensure botanical authenticity and eliminate potential variability arising from mixed-origin materials, molecular identification was performed using DNA barcoding analysis. Genomic DNA was extracted from SCG samples and amplified targeting standard plant barcode regions, followed by sequencing and comparison against reference databases. The authentication and taxonomic identification were conducted at the Molecular Biology and Genomics Laboratory, Universitas Islam Negeri (UIN) Sunan Kalijaga Yogyakarta, Indonesia.

All solvents used for extraction and chromatographic analysis, including methanol, ethanol, and acetonitrile (LC–MS grade), were purchased from Merck (Darmstadt, Germany), while formic acid (≥98%) was obtained from Sigma-Aldrich (St. Louis, MO, USA). Reference standards such as chlorogenic acid were procured from Cayman Chemical (Ann Arbor, MI, USA). Biochemical assay kits for serum creatinine and blood urea nitrogen (BUN) were obtained from Abcam (Cambridge, UK), while oxidative stress markers including malondialdehyde (MDA) and superoxide dismutase (SOD) activity were measured using kits from Elabscience (Wuhan, China) and Cayman Chemical (Ann Arbor, MI, USA), respectively. Enzyme-linked immunosorbent assay (ELISA) kits for NF-κB p65, transforming growth factor beta 1 (TGF-β1), and nuclear factor erythroid 2-related factor 2 (Nrf2) were purchased from Cloud-Clone Corp. (Wuhan, China), whereas inflammatory cytokines TNF-α and IL-6 were quantified using kits from R&D Systems (Minneapolis, MN, USA). Indoxyl sulfate was measured using an ELISA kit from MyBioSource (San Diego, CA, USA), and analytical standards for short-chain fatty acids (SCFAs) were obtained from Sigma-Aldrich (St. Louis, MO, USA). All reagents were of analytical grade or higher and used without further purification.

### 2.2. Bioactivity-Guided Extraction and Fractionation

SCG powder was subjected to bioactivity-guided extraction using 70% ethanol (solid-to-solvent ratio 1:10, *w*/*v*) under ultrasonic-assisted extraction (UAE) conditions (40 kHz, 30 min) using an ultrasonic bath (Branson Ultrasonics, Danbury, CT, USA) [14]. The extract was filtered through Whatman No. 1 filter paper and concentrated under reduced pressure using a rotary evaporator (Buchi Rotavapor R-300, Flawil, Switzerland) at 40 °C to obtain a crude extract. Fractionation was performed by open-column chromatography using silica gel 60 (70–230 mesh, Merck, Darmstadt, Germany) as the stationary phase. The crude extract was sequentially eluted using a stepwise gradient of n-hexane:ethyl acetate (100:0, 80:20, 60:40, 40:60, 20:80, and 0:100, *v*/*v*) followed by ethyl acetate:methanol (90:10, 70:30, and 50:50, *v*/*v*). Eluates with similar thin-layer chromatography (TLC) profiles were pooled, yielding eight major fractions (F1–F8). Each fraction was evaluated for total phenolic content (Folin–Ciocalteu assay), DPPH radical-scavenging activity, and preliminary anti-inflammatory activity. The fraction exhibiting the highest phenolic content together with the strongest antioxidant and anti-inflammatory activities was selected for subsequent chemical characterization and biological evaluation. The yield and chromatographic profile of the selected fraction were recorded.

### 2.3. Chemical Profiling (UHPLC–HRMS Analysis)

Comprehensive metabolite profiling of the selected polyphenol fraction was conducted using ultra-high-performance liquid chromatography coupled with high-resolution mass spectrometry (UHPLC-HRMS) (Vanquish Horizon UHPLC system coupled to Orbitrap Exploris 240, Thermo Fisher Scientific, Waltham, MA, USA) [15]. Separation was achieved using an Accucore Phenyl Hexyl column (100 × 2.1 mm, 2.6 µm; Thermo Fisher Scientific) maintained at 40 °C. The mobile phase consisted of water containing 0.1% formic acid (A) and acetonitrile (B), with a gradient elution program from 5% to 90% B over 16 min at a flow rate of 0.3 mL/min. Mass spectrometric detection was performed in both positive and negative electrospray ionization modes, with full-scan acquisition over m/z 100–1500 at a resolving power of 60,000. Data-dependent MS/MS fragmentation was performed for structural elucidation. Metabolite annotation was based on comparisons with the mzCloud and METLIN databases using a mass accuracy threshold of ≤5 ppm, agreement between experimental and reference MS/MS fragmentation spectra (similarity score ≥80%), and consistency of retention behavior with the proposed compound class. Compound identification was assigned according to the Metabolomics Standards Initiative (MSI) guidelines, with most metabolites reported as Level 2 (putatively annotated compounds) based on high-confidence spectral matching and accurate mass measurements [16].

### 2.4. In Silico Molecular Docking Analysis

In silico molecular docking was conducted targeting key proteins involved in hypertension-associated kidney injury, including NF-κB, Keap1/Nrf2 complex, TGF-β receptor, angiotensin-converting enzyme (ACE), and angiotensin II type 1 receptor (AT1). Protein structures were retrieved from the Protein Data Bank (PDB) and prepared by removing water molecules and adding polar hydrogens using AutoDock Tools 1.5.6 (The Scripps Research Institute, La Jolla, CA, USA). Ligand structures, including chlorogenic acid derivatives, were drawn using ChemDraw 25 (PerkinElmer, Waltham, MA, USA) and energy-minimized using the MMFF94 force field. Docking simulations were performed using AutoDock Vina, and binding affinities (kcal/mol) were recorded. Interaction analysis, including hydrogen bonds and hydrophobic interactions, was visualized using Discovery Studio Visualizer (Dassault Systèmes, San Diego, CA, USA), and the best binding poses were selected based on lowest binding energy and interaction stability [17,18].

### 2.5. In Vitro Cell-Based Bioactivity Assessment

The in vitro bioactivity of SCG crude extract and polyphenol-enriched fraction was evaluated using two complementary cell models representing renal tubular injury and endothelial dysfunction. Human kidney proximal tubular epithelial cells (HK-2; American Type Culture Collection, Manassas, VA, USA) and human umbilical vein endothelial cells (HUVECs; Lonza, Basel, Switzerland) were cultured under standard conditions at 37 °C in a humidified atmosphere containing 5% CO_2_. HK-2 cells were exposed to angiotensin II (1 µM) and H_2_O_2_ (200 µM) for 24 h to induce oxidative renal tubular injury, whereas HUVECs were treated with L-NAME (1 mM) and TNF-α (10 ng/mL) for 24 h to establish an endothelial dysfunction model. Following injury induction, cells were treated with SCG crude extract or the polyphenol-enriched fraction (25 or 50 µg/mL) for 24 h under identical culture conditions. HK-2 cells were maintained in Dulbecco’s Modified Eagle Medium/F12 (DMEM/F12; Gibco, Thermo Fisher Scientific, Waltham, MA, USA), while HUVECs were maintained in endothelial growth medium (EGM-2; Lonza, Basel, Switzerland), both supplemented with 10% fetal bovine serum (FBS; Gibco, Thermo Fisher Scientific, Waltham, MA, USA), 1% penicillin–streptomycin (Gibco, Thermo Fisher Scientific, Waltham, MA, USA), and appropriate growth supplements. Cells were seeded in 96-well plates and allowed to reach approximately 70–80% confluence before treatment. To mimic hypertension-associated renal tubular oxidative injury, HK-2 cells were exposed to a combined angiotensin II and hydrogen peroxide challenge (AngII/H_2_O_2_; Sigma-Aldrich, St. Louis, MO, USA) [19].

HUVECs were challenged with L-NAME and tumor necrosis factor-alpha (TNF-α; R&D Systems, Minneapolis, MN, USA) [20]. Following injury induction, cells were treated with SCG crude extract or SCG polyphenol-enriched fraction at 25 and 50 µg/mL. Untreated cells served as normal controls, while injured untreated cells served as injury controls. Cell viability was assessed using a colorimetric MTT assay kit (Abcam, Cambridge, UK) or equivalent tetrazolium-based viability assay according to the manufacturer’s protocol, with absorbance measured using a microplate reader (BioTek Synergy HTX Multi-Mode Reader, Agilent BioTek Instruments, Winooski, VT, USA) [21]. Intracellular reactive oxygen species (ROS) generation was quantified using a DCFH-DA fluorescent probe (Sigma-Aldrich, St. Louis, MO, USA), and fluorescence intensity was expressed as percentage of the normal control. NF-κB, TGF-β1, and Nrf2 activities or expression levels were quantified using commercially available ELISA-based assay kits (Cloud-Clone Corp., Wuhan, China) to determine the inflammatory, fibrotic, and antioxidant signaling responses. Nitric oxide (NO) bioavailability, particularly in HUVECs, was measured using a Griess reagent-based nitric oxide assay kit (Cayman Chemical, Ann Arbor, MI, USA), with results expressed as percentage of control. All cell culture experiments were performed using three independent biological replicates, with three technical replicates analyzed for each treatment condition. Data are presented as the mean ± SD of the biological replicates. All in vitro outcomes were normalized to the untreated control group and expressed as percentage values, including cell viability, ROS production, NF-κB, TGF-β1, Nrf2, and NO levels.

### 2.6. In Vivo Experimental Animals and Study Design

**Figure 1:**
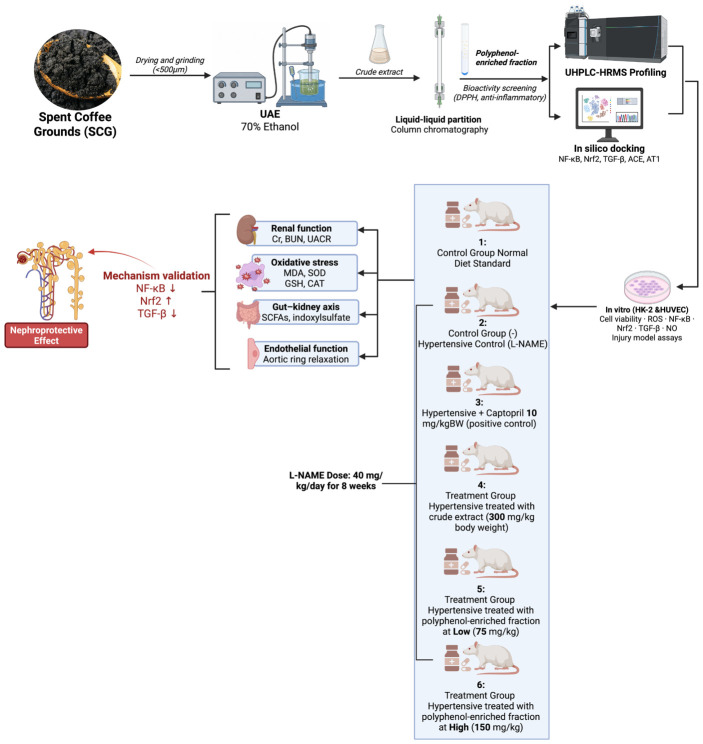
Experimental workflow and study design of SCG-derived polyphenol fraction in hypertension-associated kidney injury. Created in BioRender. Nurkolis, F. (2026) https://BioRender.com/ckicpzz.

Male Wistar rats (*Rattus norvegicus*) weighing 180–220 g were obtained from an accredited laboratory animal facility and acclimatized for one week under controlled environmental conditions (22 ± 2 °C, 55 ± 10% humidity, 12 h light/dark cycle). Animals were provided standard laboratory chow and water ad libitum. Animals were randomly assigned to six experimental groups (*n* = 10 per group). Based on a significance level of 0.05, a statistical power of 80%, and a large expected effect size (Cohen’s f = 0.50), the minimum sample size required was eight animals per group. To account for possible experimental losses and to preserve statistical power throughout the study, ten animals were included in each group. Hypertension-associated kidney injury was induced by oral administration of Nω-nitro-L-arginine methyl ester (L-NAME; Sigma-Aldrich, St. Louis, MO, USA) at a dose of 40 mg/kg/day for 8 weeks [22]. After 2 weeks of hypertension induction with L-NAME, animals received the extract treatment for the subsequent 6 weeks. Following induction, rats were randomly assigned into six experimental groups, normal control (Standard diet without hypertension), hypertensive control (L-NAME), hypertensive treated with captopril as a positive control, hypertensive treated with crude extract (300 mg/kg body weight (BW)), and hypertensive treated with polyphenol-enriched fraction at low (75 mg/kgBW) and high (150 mg/kgBW) doses. Captopril (Sigma-Aldrich, St. Louis, MO, USA) was administered orally at a dose of 10 mg/kg body weight/day as a positive control, based on its established efficacy in L-NAME-induced hypertension and renal injury models. All experimental procedures were conducted in accordance with institutional animal care guidelines and were approved by the Health Research Ethics Committee of the Faculty of Medicine, Brawijaya University (Ethical Approval No. 203/EC/KEPK/05/2025). The experimental workflow and study design are summarized in Figure 1.

#### 2.6.1. Dose Selection Rationale

The doses of SCG crude extract and polyphenol-enriched fraction were selected based on a potency-adjusted, bioactivity-guided dosing strategy (Figure 1). The crude extract was administered at 300 mg/kg body weight. In contrast, the polyphenol-enriched fraction was administered at lower doses of 75 mg/kg and 150 mg/kg body weight, representing low- and high-dose treatments, respectively, particularly chlorogenic acid derivatives and related phenolic compounds. The 75 mg/kg dose was selected as an initial effective dose to evaluate biological activity with reduced extract burden, whereas the 150 mg/kg dose was chosen as a two-fold escalation to assess dose-dependent efficacy while remaining within a commonly used and well-tolerated dosing range for plant-derived polyphenol fractions in rodent models. Captopril was administered at a dose of 10 mg/kg body weight/day as a positive control based on its well-established efficacy in L-NAME-induced hypertension and renal injury models.

#### 2.6.2. Hemodynamic and Renal Function Measurements

Systolic blood pressure (SBP) was measured weekly using a non-invasive tail-cuff system (CODA, Kent Scientific Corporation, Torrington, CT, USA), with animals acclimatized prior to measurement to reduce stress-induced variability [23]. Blood samples were collected via cardiac puncture under anesthesia, and serum was separated by centrifugation at 3000 rpm for 10 min. Serum creatinine and BUN levels were measured using colorimetric assay kits (Abcam, Cambridge, UK), while urinary albumin–creatinine ratio (UACR) was quantified using ELISA-based methods according to the manufacturer’s instructions.

#### 2.6.3. Oxidative Stress, Inflammation, and Fibrosis Markers

Renal tissues were homogenized in cold phosphate-buffered saline and centrifuged to obtain supernatants for biochemical analysis. Lipid peroxidation was assessed by measuring MDA levels using a thiobarbituric acid reactive substances (TBARS) assay kit (Elabscience, Wuhan, China), while antioxidant defense was evaluated via SOD activity assay (Cayman Chemical, Ann Arbor, MI, USA) [24]. Inflammatory cytokines TNF-α and IL-6 were quantified using ELISA kits (R&D Systems, Minneapolis, MN, USA). Key signaling molecules including NF-κB p65, TGF-β1, and Nrf2 were measured using ELISA kits (Cloud-Clone Corp., Wuhan, China) to assess inflammatory, fibrotic, and antioxidant pathways.

#### 2.6.4. Gut–Kidney Axis and Endothelial Function Assessment

Serum indoxyl sulfate levels were determined using a competitive ELISA kit (MyBioSource, San Diego, CA, USA), reflecting gut-derived uremic toxin burden. Fecal SCFAs were extracted using acidified solvents and quantified via gas chromatography (Agilent 7890B GC system, Agilent Technologies, Santa Clara, CA, USA). Endothelial function was assessed using isolated thoracic aortic ring preparations mounted in an organ bath system (Danish Myo Technology, Aarhus, Denmark), where vasorelaxation responses to acetylcholine were recorded following pre-contraction with phenylephrine [25].

#### 2.6.5. Microbiome Analysis and Statistical Evaluation

Gut microbiota composition was analyzed using 16S rRNA gene sequencing targeting the V3–V4 region with primers 338F/806R on an Illumina MiSeq platform (Illumina Inc., San Diego, CA, USA) [26]. Sequence data were processed using QIIME2, and operational taxonomic units (OTUs) were assigned at 97% similarity against the Silva database. Statistical analyses were performed using GraphPad Prism 10.6.1 (GraphPad Software, San Diego, CA, USA). Data are presented as mean ± standard deviation (SD). Prior to statistical analysis, data distribution was evaluated using the Shapiro–Wilk normality test, and homogeneity of variance was assessed using Levene’s test. When both assumptions were satisfied, differences among groups were analyzed using one-way analysis of variance (ANOVA) followed by Tukey’s multiple-comparison post hoc test. If the assumptions of normality or homogeneity of variance were not met, data were analyzed using the Kruskal–Wallis test followed by Dunn’s multiple-comparison test. A two-sided *p* < 0.05 was considered statistically significant.

#### 2.6.6. Targeted Metabolomic Analysis of Gut–Kidney Axis-Related Metabolites

Targeted metabolomic analysis was performed to quantify gut–kidney axis-related metabolites, including short-chain fatty acids (SCFAs: butyrate, propionate, and acetate), gut-derived uremic toxins (indoxyl sulfate and p-cresyl sulfate), trimethylamine N-oxide (TMAO), kynurenine, and hippurate [27]. Fecal samples were used for SCFA analysis, whereas serum or plasma samples were used for circulating uremic toxins and host–microbial co-metabolites. For SCFA quantification, fecal samples were weighed, homogenized in acidified water containing an internal standard, vortexed, sonicated, and centrifuged to obtain clear supernatants. The supernatants were filtered through a 0.22 µm membrane filter and analyzed using gas chromatography–mass spectrometry (GC–MS) or gas chromatography with flame ionization detection (GC–FID) (Agilent Technologies, Santa Clara, CA, USA). Acetate, propionate, and butyrate were identified by comparison with authentic standards (Sigma-Aldrich, St. Louis, MO, USA) and quantified using external calibration curves, with results expressed as µmol/g feces. For indoxyl sulfate, p-cresyl sulfate, TMAO, kynurenine, and hippurate, serum samples were thawed on ice and subjected to protein precipitation using cold methanol or acetonitrile containing appropriate internal standards. After vortexing and centrifugation, the supernatants were collected, filtered, and injected into a liquid chromatography–tandem mass spectrometry system (LC–MS/MS) equipped with an electrospray ionization source (UHPLC system coupled to a triple quadrupole or high-resolution mass spectrometer; Thermo Fisher Scientific, Waltham, MA, USA). Chromatographic separation was achieved using a reversed-phase C18 column with a mobile phase consisting of water containing 0.1% formic acid and acetonitrile or methanol containing 0.1% formic acid. Metabolites were monitored in selected reaction monitoring or targeted full-scan/MS/MS mode, and concentrations were calculated based on calibration curves generated from analytical standards of indoxyl sulfate, p-cresyl sulfate, TMAO, kynurenine, and hippurate (Sigma-Aldrich, St. Louis, MO, USA). Quality control samples, blank injections, and pooled biological samples were included throughout the analytical run to monitor instrument stability, carryover, retention time reproducibility, and analytical precision. Metabolite concentrations were normalized to sample weight for fecal SCFAs or serum volume for circulating metabolites. This targeted metabolomic approach was used to evaluate whether SCG-derived polyphenol fraction modulated microbial fermentation products, uremic toxin burden, and host–microbial metabolites associated with hypertension-related kidney injury.

## 3. Results

### 3.1. Chemical Characterization Reveals Enrichment of Bioactive Chlorogenic Acid Derivatives

**Figure 2:**
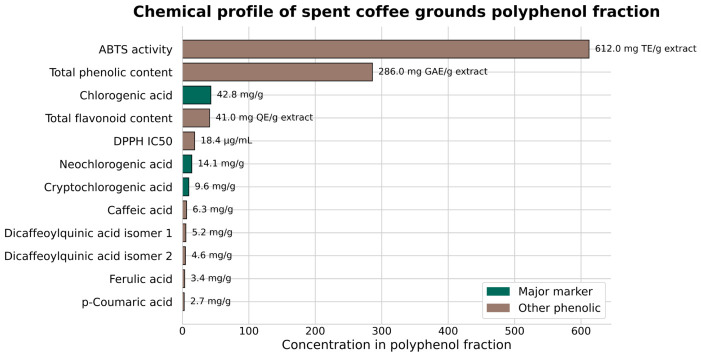
Chemical profile of SCG-derived polyphenol fraction identified by UHPLC–HRMS. The chromatographic and mass spectrometric analysis revealed enrichment of chlorogenic acid derivatives, including mono-caffeoylquinic acids (5-CQA, neochlorogenic acid, cryptochlorogenic acid) and dicaffeoylquinic acids (diCQAs), along with minor phenolics such as caffeic acid and ferulic acid. Representative compounds were selected based on abundance and biological relevance for subsequent in silico molecular docking analysis.

UHPLC–HRMS profiling demonstrated that the bioactivity-guided fractionation of spent coffee grounds (SCG) resulted in a distinct enrichment of polyphenolic compounds, predominantly chlorogenic acid derivatives (Figure 2). The chemical profile was characterized by the dominance of mono-caffeoylquinic acids (CQA), including 5-CQA (chlorogenic acid), neochlorogenic acid, and cryptochlorogenic acid, alongside higher-order derivatives such as dicaffeoylquinic acids (diCQAs). In addition, minor phenolics including caffeic acid and ferulic acid were consistently detected, indicating preservation of structurally diverse antioxidant constituents.

The enrichment strategy significantly altered the chemical composition toward compounds with established redox-modulating and anti-inflammatory potential. Given their relative abundance and known pharmacological relevance, representative compounds—particularly 5-CQA and diCQA isomers—were selected for downstream mechanistic interrogation through molecular docking. This targeted selection enabled a mechanistically informed transition from chemical profiling to functional prediction, linking compositional enrichment to potential biological activity.

Collectively, these findings confirm that bioactivity-guided fractionation successfully concentrates key polyphenolic drivers within SCG, establishing a chemically defined basis for subsequent multi-target pharmacological evaluation.

Based on UHPLC–HRMS profiling, the major and representative polyphenolic compounds identified, particularly chlorogenic acid derivatives (5-CQA, neochlorogenic acid, cryptochlorogenic acid, and dicaffeoylquinic acids), along with selected minor phenolics (caffeic acid and ferulic acid), were chosen for in silico molecular docking analysis due to their abundance and known biological relevance.

### 3.2. In Silico Target Prediction Supports Multi-Pathway Modulation

To explore potential molecular interactions underlying the observed biological effects, molecular docking analysis was performed against key proteins implicated in hypertension-associated kidney injury, including NF-κB, the Keap1/Nrf2 complex, TGF-β receptor I, angiotensin-converting enzyme (ACE), and the angiotensin II type 1 (AT1) receptor (Table 1).

Among the evaluated compounds, 5-CQA exhibited strong and consistent binding across multiple targets, demonstrating high affinity toward NF-κB p65 (−8.4 kcal/mol), TGF-β receptor I (−8.1 kcal/mol), and the Keap1 Kelch domain (−8.7 kcal/mol). These predicted interactions suggest that chlorogenic acid derivatives may influence inflammatory, antioxidant, and fibrotic signaling pathways, providing a plausible molecular basis for the biological effects observed in the experimental models.

Dicaffeoylquinic acid derivatives further extended this multi-target profile into the hemodynamic axis. diCQA-1 showed the strongest binding to ACE (−9.0 kcal/mol), exceeding the reference inhibitor captopril (−8.0 kcal/mol), while diCQA-2 demonstrated comparable affinity to the AT1 receptor (−8.5 kcal/mol), matching the standard antagonist losartan. These findings suggest the potential for interaction with components of the RAAS pathway, although direct inhibition requires experimental confirmation.

In contrast, minor phenolics such as caffeic acid and ferulic acid exhibited moderate binding affinities (−6.9 and −7.1 kcal/mol, respectively), suggesting supportive but less dominant contributions to the overall pharmacological profile.

Taken together, the docking results support the possibility that SCG-derived polyphenols interact with multiple molecular targets relevant to hypertension-associated kidney injury. These computational predictions are consistent with the observed in vitro and in vivo findings but should be interpreted as hypothesis-generating rather than definitive evidence of pathway modulation.

### 3.3. In Vitro Cytoprotection and Signaling Modulation Across Renal and Endothelial Models

**Figure 3:**
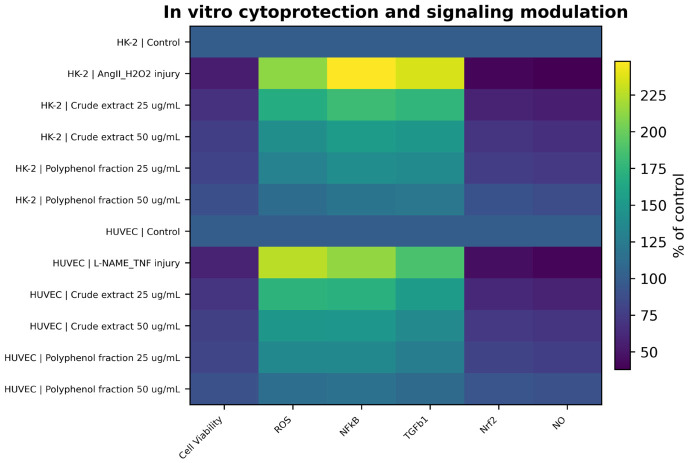
Integrated heatmap of in vitro cytoprotection and signaling modulation in HK-2 and HUVEC models. Heatmap values are expressed as percentage of control, illustrating relative changes in cell viability, ROS, NF-κB, TGF-β1, Nrf2, and NO across treatment conditions. Injury models (HK-2: AngII/H_2_O_2_; HUVEC: L-NAME/TNF-α) show increased oxidative stress, inflammation, and fibrosis markers with reduced viability and NO bioavailability. Treatment with SCG crude extract and polyphenol-enriched fraction demonstrates dose-dependent normalization of these parameters, with the polyphenol fraction exhibiting superior efficacy.

The integrated heatmap analysis revealed a consistent injury-associated shift in both renal tubular (HK-2) and endothelial (HUVEC) models, characterized by reduced cell viability and nitric oxide (NO) bioavailability, accompanied by marked elevations in ROS, NF-κB, and TGF-β1, alongside suppression of Nrf2 signaling (Figure 3).

In HK-2 cells, AngII/H_2_O_2_ exposure induced a pronounced cytotoxic and pro-oxidative profile, with cell viability decreasing to approximately 60% of control, while ROS, NF-κB, and TGF-β1 increased to ~200–240% of control levels. These alterations were partially mitigated by crude extract treatment; however, the polyphenol-enriched fraction demonstrated a more substantial and dose-dependent normalization of these parameters. At 50 µg/mL, the polyphenol fraction restored cell viability close to baseline (~85–90%), while markedly suppressing ROS and inflammatory/fibrotic signaling toward near-control levels. Notably, Nrf2 activity was concurrently elevated, indicating recovery of antioxidant defense capacity.

A similar pattern was observed in HUVECs subjected to L-NAME/TNF-α-induced endothelial dysfunction. Injury conditions resulted in substantial oxidative and inflammatory activation, with ROS and NF-κB exceeding ~200% of control, accompanied by reduced NO bioavailability (~50–60%). Treatment with the polyphenol fraction effectively reversed these alterations, restoring NO levels and suppressing ROS, NF-κB, and TGF-β1 in a dose-dependent manner. Again, the polyphenol fraction consistently outperformed the crude extract across all measured endpoints.

Importantly, the parallel response patterns across HK-2 and HUVEC models highlight a coordinated dual-cellular protective mechanism, targeting both renal epithelial integrity and endothelial function. This convergence supports a systemic mode of action involving redox rebalancing and suppression of inflammation and fibrosis, consistent with the proposed NF-κB/Nrf2/TGF-β regulatory axis.

### 3.4. Polyphenol Fraction Restores Hemodynamic Stability and Multi-Compartment Renal Integrity

**Figure 4:**
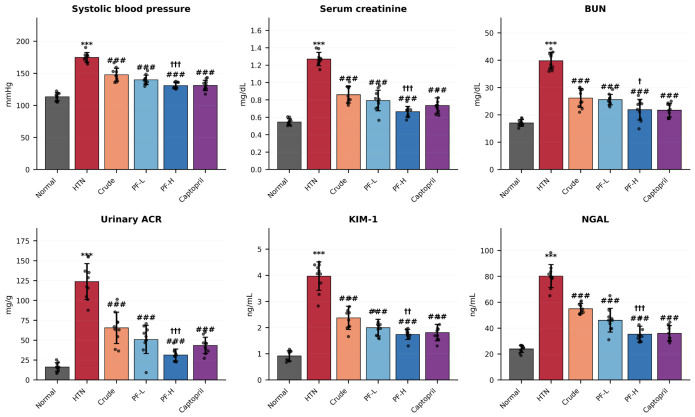
Effects of SCG polyphenol fraction on hemodynamic and renal injury parameters in L-NAME-induced hypertensive rats. Hypertension significantly increased systolic blood pressure (SBP), serum creatinine, blood urea nitrogen (BUN), urinary albumin–creatinine ratio (UACR), and tubular injury markers KIM-1 and NGAL. Treatment with SCG crude extract and polyphenol-enriched fraction attenuated these alterations in a dose-dependent manner, with the high-dose polyphenol fraction demonstrating effects comparable to captopril. Data are presented as mean ± SD (*n* = 10/group). *** *p* < 0.001 vs. normal; ### *p* < 0.001 vs. HTN; † *p* < 0.05, †† *p* < 0.01, ††† *p* < 0.001 vs. crude extract (Tukey’s post hoc test).

L-NAME administration induced a pronounced hypertensive phenotype, evidenced by a significant elevation in systolic blood pressure (SBP), alongside extensive renal dysfunction and injury (Figure 4). Compared with the normal group, hypertensive rats exhibited markedly increased SBP (178.1 ± 9.6 mmHg, *p* < 0.001), accompanied by significant elevations in serum creatinine (1.30 ± 0.15 mg/dL, *p* < 0.001) and BUN (40.1 ± 3.2 mg/dL, *p* < 0.001), indicating impaired renal filtration.

Glomerular injury was further reflected by a substantial increase in urinary albumin–creatinine ratio (UACR), which rose to 125.0 ± 25.3 mg/g (*p* < 0.001). In parallel, tubular damage markers were significantly elevated, with KIM-1 reaching 4.0 ± 0.5 ng/mL (*p* < 0.001) and NGAL increasing to 80.1 ± 8.2 ng/mL (*p* < 0.001), indicating severe tubular injury.

Treatment with SCG-derived polyphenol fraction markedly attenuated these alterations in a dose-dependent manner. The high-dose group (PF-H) significantly reduced SBP to 125.1 ± 9.0 mmHg (*p* < 0.001 vs. HTN), approaching the effect of captopril. Renal function markers were concurrently improved, with serum creatinine and BUN decreasing to 0.67 ± 0.05 mg/dL and 22.0 ± 3.0 mg/dL, respectively (*p* < 0.001).

Importantly, both glomerular and tubular injury markers were substantially ameliorated. UACR was reduced to 40.0 ± 10.2 mg/g, while KIM-1 and NGAL decreased to 1.8 ± 0.2 ng/mL and 35.0 ± 5.0 ng/mL, respectively (*p* < 0.001 vs. HTN). Across all endpoints, the polyphenol fraction demonstrated superior efficacy compared to the crude extract, with consistent improvements across hemodynamic, filtration, and tubular injury parameters.

Collectively, these findings indicate that the SCG polyphenol fraction confers comprehensive renoprotection across hemodynamic, glomerular, and tubular compartments, highlighting its multi-target therapeutic potential.

### 3.5. Polyphenol Fraction Attenuates Oxidative Stress, Inflammation, and Fibrotic Signaling in the Kidney

**Figure 5:**
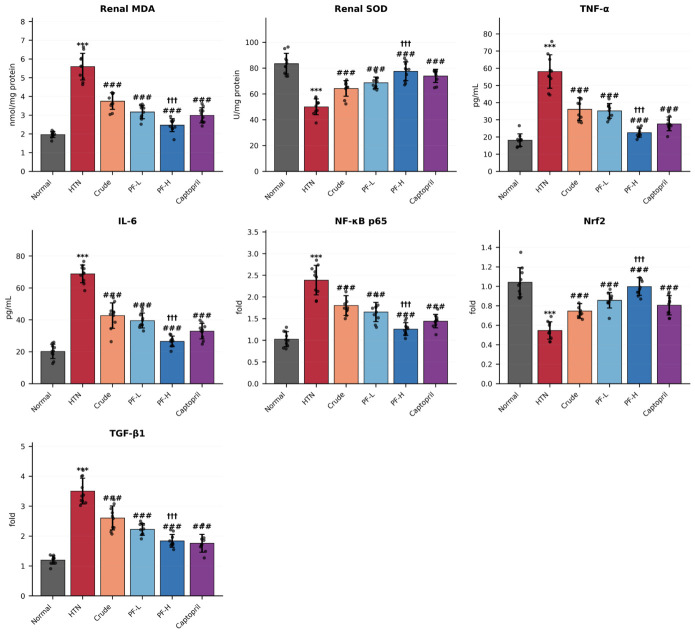
Effects of SCG polyphenol fraction on renal oxidative stress, inflammation, and fibrotic signaling pathways. Hypertension significantly increased lipid peroxidation (MDA), pro-inflammatory cytokines (TNF-α and IL-6), NF-κB p65 expression, and TGF-β1 levels, while reducing antioxidant defense (SOD activity and Nrf2 expression). Treatment with SCG crude extract and polyphenol-enriched fraction significantly reversed these alterations in a dose-dependent manner, with the high-dose polyphenol fraction showing the strongest effect and approaching captopril efficacy. Data are presented as mean ± SD (*n* = 10/group). *** *p* < 0.001 vs. normal; ### *p* < 0.001 vs. HTN; ††† *p* < 0.001 vs. crude extract (Tukey’s post hoc test).

Hypertension induced by L-NAME resulted in a pronounced imbalance in renal redox status, inflammatory activation, and profibrotic signaling (Figure 5). This was evidenced by a significant increase in lipid peroxidation, as indicated by elevated renal MDA levels (~5.5–6.0 nmol/mg protein, *p* < 0.001 vs. normal), accompanied by a marked reduction in antioxidant defense, reflected by decreased SOD activity (~50 U/mg protein, *p* < 0.001).

Concurrently, hypertensive rats exhibited robust inflammatory activation, with TNF-α and IL-6 levels significantly increased to approximately 55–60 pg/mL and 65–70 pg/mL, respectively (*p* < 0.001 vs. normal). This pro-inflammatory state was further supported by upregulation of NF-κB p65 expression (~2.3–2.5 fold, *p* < 0.001), indicating activation of canonical inflammatory signaling pathways.

In parallel, antioxidant signaling was markedly suppressed, as evidenced by reduced Nrf2 expression (~0.5–0.6 fold, *p* < 0.001), while profibrotic signaling was significantly enhanced, with TGF-β1 expression increasing to approximately 3.5–4.0 fold (*p* < 0.001).

Treatment with SCG-derived polyphenol fraction effectively reversed these alterations in a dose-dependent manner. The high-dose group (PF-H) significantly reduced MDA levels (~2.5 nmol/mg protein, *p* < 0.001 vs. HTN) while restoring SOD activity (~75–80 U/mg protein, *p* < 0.001), indicating substantial attenuation of oxidative stress.

Inflammatory markers were similarly suppressed, with TNF-α and IL-6 reduced to approximately 25–30 pg/mL and 30–35 pg/mL, respectively (*p* < 0.001 vs. HTN). This was accompanied by significant downregulation of NF-κB p65 (~1.2–1.4 fold, *p* < 0.001).

Importantly, antioxidant defense was restored, with Nrf2 expression increasing to near-normal levels (~1.0 fold, *p* < 0.001 vs. HTN), while TGF-β1 expression was markedly reduced (~1.7–2.0 fold, *p* < 0.001), indicating suppression of fibrotic signaling.

Across all parameters, the polyphenol fraction demonstrated greater efficacy than the crude extract, consistently showing stronger normalization of oxidative, inflammatory, and fibrotic markers. Collectively, these findings indicate that the SCG polyphenol fraction exerts a coordinated multi-pathway regulatory effect, simultaneously suppressing oxidative stress and inflammation while restoring antioxidant signaling and attenuating fibrosis.

### 3.6. SCG Polyphenol Fraction Remodels Gut–Kidney Axis Metabolites

Hypertension induced by L-NAME resulted in a marked reduction of short-chain fatty acids (SCFAs: butyrate, propionate, and acetate) and a concomitant increase in uremic toxins (indoxyl sulfate and p-cresyl sulfate) and host–microbial co-metabolites (TMAO and kynurenine), along with decreased hippurate levels (Table 2). Treatment with SCG crude extract and polyphenol-enriched fraction restored SCFA levels and reduced uremic toxin accumulation in a dose-dependent manner, with the high-dose polyphenol fraction showing the strongest normalization effect and approaching captopril efficacy. Data are expressed as fold change relative to normal control.

Treatment with the polyphenol fraction significantly restored SCFA levels in a dose-dependent manner, with the high-dose group approaching normal control levels. Concurrently, substantial reductions in indoxyl sulfate and p-cresyl sulfate were observed. Additional host–microbial metabolites, including trimethylamine N-oxide (TMAO) and kynurenine, were also reduced, while hippurate levels increased, indicating improved microbial metabolic function. These results demonstrate that the SCG polyphenol fraction effectively modulates gut–kidney axis metabolites, reflecting improved host–microbiome metabolic homeostasis.

### 3.7. SCG Polyphenol Fraction Preserves Renal Architecture and Restores Endothelial Function

**Figure 6:**
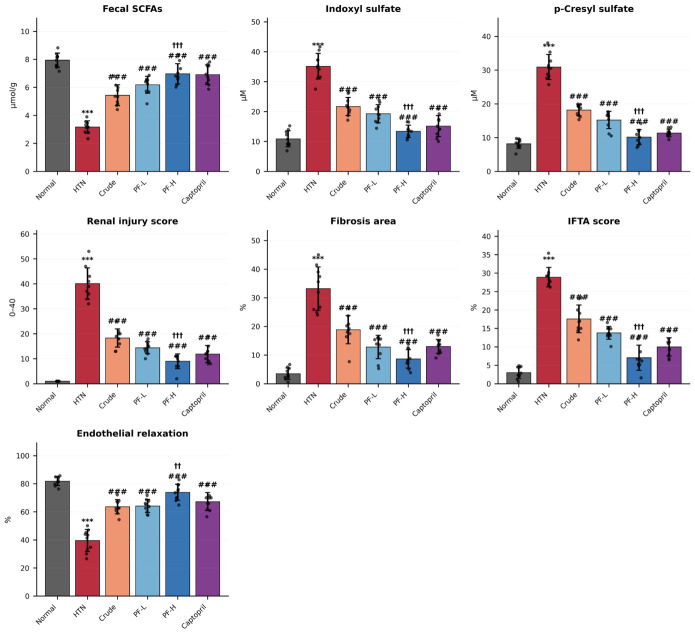
Effects of SCG polyphenol fraction on renal histopathology and endothelial function in L-NAME-induced hypertensive rats. Histological analysis shows that hypertension induced marked renal damage, including tubular degeneration, interstitial inflammation, and fibrosis, reflected by increased injury and IFTA scores. Treatment with SCG crude extract and polyphenol-enriched fraction attenuated these structural abnormalities in a dose-dependent manner, with the high-dose polyphenol fraction showing near-normal renal architecture. Endothelial function, assessed by acetylcholine-induced vasorelaxation, was significantly impaired in hypertensive rats and markedly restored following treatment. Data are presented as mean ± SD (*n* = 10/group). *** *p* < 0.001 vs. normal; ### *p* < 0.001 vs. HTN; †† *p* < 0.01, ††† *p* < 0.001 vs. crude extract (Tukey’s post hoc test).

Histopathological evaluation revealed severe structural damage in hypertensive rats, characterized by tubular degeneration, interstitial inflammation, and increased fibrosis (Figure 6). These alterations were reflected in elevated tubular injury and interstitial fibrosis/tubular atrophy (IFTA) scores, indicating progressive renal structural deterioration.

In contrast, treatment with the SCG-derived polyphenol fraction markedly attenuated these histological abnormalities in a dose-dependent manner. The crude extract partially improved renal morphology; however, the polyphenol-enriched fraction demonstrated substantially greater protective effects. In the high-dose group (PF-H), renal architecture was largely preserved, with reduced tubular injury, minimal inflammatory infiltration, and significant attenuation of fibrotic features, approaching the normal control profile.

Consistent with structural improvement, endothelial function was significantly impaired in hypertensive animals, as evidenced by reduced vasorelaxation response to acetylcholine. Hypertension decreased endothelial-dependent relaxation to approximately 40.0 ± 4.6%, indicating marked endothelial dysfunction.

Treatment with the polyphenol fraction significantly restored vascular responsiveness in a dose-dependent manner. The high-dose group exhibited a pronounced increase in vasorelaxation to 75.7 ± 5.0% (*p* < 0.001 vs. HTN), approaching the effect observed with captopril and indicating substantial recovery of endothelial function.

Importantly, the parallel improvement in renal histology and vascular function suggests a coordinated protective mechanism linking structural renoprotection with restoration of endothelial integrity. These findings support the concept that SCG polyphenol fraction exerts multi-organ protective effects across the kidney–vascular axis, reinforcing its systemic therapeutic potential.

### 3.8. Microbiome Remodeling and Diversity Restoration

**Figure 7:**
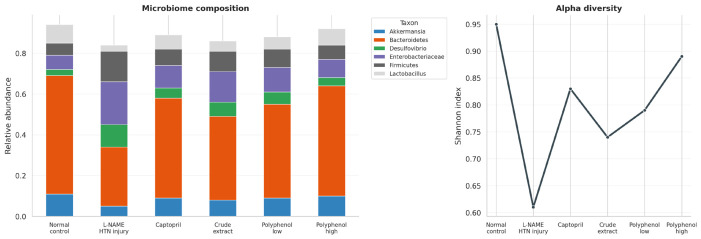
Gut microbiome composition and alpha diversity across experimental groups. Stacked bar plots illustrate the relative abundance of key bacterial taxa, including *Akkermansia*, *Bacteroidetes*, *Desulfovibrio*, *Enterobacteriaceae*, *Firmicutes*, and *Lactobacillus*. The alpha diversity plot (Shannon index) shows reduced microbial diversity in the hypertensive group compared with the normal control, indicating dysbiosis. Treatment with SCG crude extract and polyphenol-enriched fraction partially restored microbial diversity and shifted the taxonomic profile toward a more balanced composition, with the high-dose polyphenol group showing the closest resemblance to the normal control. Data are presented as mean values for descriptive comparison.

Gut microbiome profiling revealed a clear shift in community composition and diversity across experimental groups (Figure 7). Hypertension induced by L-NAME was associated with a marked alteration in microbial structure, characterized by a reduction in alpha diversity and a shift toward a dysbiotic profile.

Specifically, the hypertensive group exhibited a lower Shannon index compared with the normal control, indicating reduced microbial richness and evenness. This decrease in diversity was accompanied by a compositional imbalance, with an apparent increase in taxa associated with dysbiosis, including *Enterobacteriaceae* and *Desulfovibrio*, and a relative reduction in beneficial bacteria such as *Akkermansia* and *Lactobacillus*.

Treatment with SCG-derived interventions resulted in a gradual restoration of microbial diversity and composition. The polyphenol-enriched fraction, particularly at the high dose (PF-H), demonstrated the most pronounced recovery, with Shannon index values approaching those observed in the normal control group. This suggests a re-establishment of microbial ecological balance.

At the compositional level, treatment groups showed a relative enrichment of beneficial taxa, including *Akkermansia* and *Lactobacillus*, alongside a reduction in potentially harmful taxa such as *Enterobacteriaceae* and *Desulfovibrio*. These shifts were more evident in the polyphenol fraction groups compared with the crude extract, indicating a stronger modulatory effect on the gut microbiome.

Overall, these findings indicate that the SCG polyphenol fraction promotes microbiome rebalancing, characterized by improved diversity and a shift toward a more beneficial microbial profile, which is consistent with the observed improvements in gut–kidney axis metabolites and renal outcomes. Notably, the restoration of microbial diversity closely paralleled the normalization of SCFA production and reduction of uremic toxins, suggesting a functional linkage between microbiome structure and metabolic output.

### 3.9. Microbiome Remodeling Signature and Taxonomic Rebalancing

**Figure 8:**
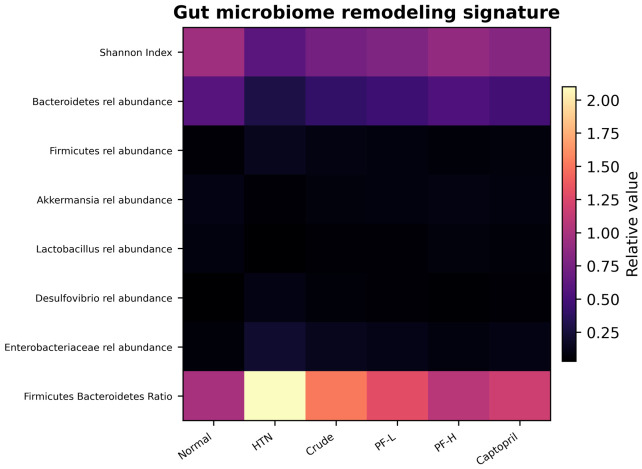
Heatmap of gut microbiome remodeling signature across experimental groups. The heatmap illustrates relative changes in alpha diversity (Shannon index), key bacterial taxa abundance (*Bacteroidetes*, *Firmicutes*, *Akkermansia*, *Lactobacillus*, *Desulfovibrio*, *Enterobacteriaceae*), and the *Firmicutes*/*Bacteroidetes* ratio. Hypertension (HTN) induced a dysbiotic profile characterized by reduced microbial diversity and an elevated F/B ratio, along with increased abundance of pro-inflammatory taxa. Treatment with SCG crude extract and polyphenol-enriched fraction progressively restored microbial balance, with the high-dose polyphenol group (PF-H) showing the closest resemblance to the normal control. Color intensity represents relative abundance or normalized values.

Heatmap-based profiling revealed a distinct microbiome remodeling signature across experimental groups, highlighting coordinated shifts in microbial diversity and taxonomic composition (Figure 8). Hypertension markedly disrupted gut microbial homeostasis, as evidenced by a reduction in the Shannon diversity index and a pronounced increase in the Firmicutes/Bacteroidetes (F/B) ratio. This imbalance is a well-recognized hallmark of dysbiosis and metabolic dysfunction. Concomitantly, hypertensive animals exhibited reduced relative abundance of beneficial taxa, including *Akkermansia* and *Lactobacillus*, alongside an enrichment of potentially pathogenic and pro-inflammatory bacteria such as *Enterobacteriaceae* and *Desulfovibrio*.

Intervention with SCG-derived treatments induced a progressive normalization of this dysbiotic profile. Notably, the polyphenol-enriched fraction demonstrated a dose-dependent effect, with the high-dose group (PF-H) showing the most pronounced microbiome rebalancing. This was reflected by an increase in the Shannon index, indicating improved microbial diversity, and a substantial reduction in the F/B ratio toward levels observed in the normal control.

At the taxonomic level, the polyphenol fraction promoted the enrichment of beneficial bacteria, particularly *Akkermansia* and *Lactobacillus*, which are associated with enhanced mucosal integrity and short-chain fatty acid production. Simultaneously, there was a marked suppression of *Enterobacteriaceae* and *Desulfovibrio*, indicating attenuation of pro-inflammatory and toxin-producing microbial populations.

Interestingly, while crude extract treatment partially improved microbiome composition, its effects were less pronounced compared with the polyphenol-enriched fraction, supporting the hypothesis that bioactive enrichment enhances microbiome-modulatory efficacy.

Overall, the heatmap signature demonstrates that SCG polyphenol fraction induces coordinated ecological reprogramming of the gut microbiome, restoring diversity and shifting the microbial landscape toward a metabolically favorable and anti-inflammatory state.

## 4. Discussion

**Figure 9:**
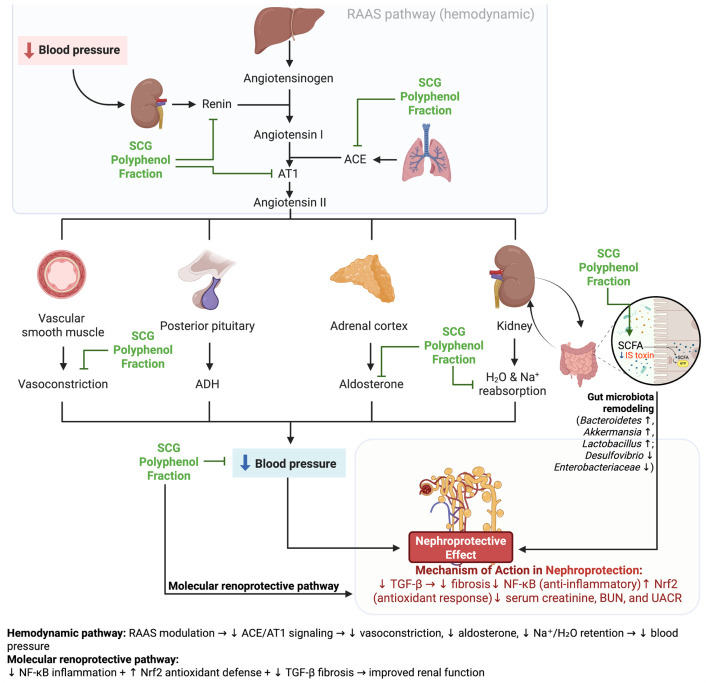
Proposed hypothetical mechanism underlying the nephroprotective effects of the SCG-derived polyphenol fraction. The schematic presents a working mechanistic hypothesis integrating the in silico, in vitro, and in vivo findings of this study. It proposes that chlorogenic acid-rich polyphenols may contribute to nephroprotection through modulation of NF-κB-, Nrf2-, TGF-β-, and RAAS-associated pathways, together with improvements in gut–kidney axis homeostasis. The proposed interactions are hypothesis-generating and require further molecular validation. Created in BioRender. Nurkolis, F. (2026) https://BioRender.com/lhne27y.

The present study demonstrates that a bioactivity-guided polyphenol fraction derived from SCG exerts robust nephroprotective effects in hypertension-associated kidney injury through coordinated modulation of oxidative stress, inflammation, fibrosis, and gut–kidney axis signaling (Figure 9). A central finding of this study is the dominant role of chlorogenic acid derivatives as key bioactive constituents. UHPLC–HRMS analysis identified high levels of chlorogenic acid (42.8 mg/g) and related isomers, which aligns with previous reports describing coffee-derived polyphenols as potent modulators of metabolic and inflammatory processes [28,29]. These compounds are known to possess strong redox-modulating properties and may act as upstream regulators of multiple signaling pathways, thereby explaining the enhanced biological effects observed in the fraction-treated groups [30].

The in silico docking results further support a multi-target mechanism of action, with strong binding affinities observed for NF-κB, Keap1, TGF-β receptor, ACE, and AT1 receptor. The high predicted affinity of 5-CQA for Keap1 (−8.7 kcal/mol) is consistent with a potential interaction that may contribute to Nrf2 activation [31]. However, molecular docking provides computational evidence of binding propensity rather than direct confirmation of target engagement or disruption of the Keap1–Nrf2 complex under biological conditions. Therefore, these findings should be interpreted as mechanistic hypotheses that are supported by, but not definitive proof of, the observed biological responses. The strong binding of dicaffeoylquinic acid derivatives to ACE (−9.0 kcal/mol) and AT1 receptor (−8.5 kcal/mol) also supports the possibility of an additional hemodynamic regulatory component that may contribute to the observed physiological improvements [32], although direct target engagement remains to be experimentally confirmed. Therefore, the docking analysis should be interpreted as complementary computational evidence that prioritizes plausible molecular targets rather than as direct confirmation of ligand–target binding or downstream signaling modulation.

At the cellular level, the polyphenol fraction effectively restored redox balance and suppressed inflammatory signaling in both renal tubular (HK-2) and endothelial (HUVEC) models. The marked recovery of cell viability to ~90% and reduction of ROS and NF-κB levels indicate that the fraction mitigates oxidative injury and inflammatory activation at early stages of cellular dysfunction. These findings are consistent with previous studies demonstrating that polyphenols can act as dual modulators of oxidative stress and endothelial function [33,34], although the magnitude of effect observed here appears greater due to the enriched composition. Notably, in HUVECs, the polyphenol fraction markedly restored endothelial nitric oxide (NO) bioavailability while concurrently suppressing ROS and NF-κB activation under L-NAME/TNF-α-induced stress, indicating a direct protective effect on endothelial redox homeostasis. This endothelial-targeted response is particularly relevant, as it suggests that the fraction not only preserves cellular integrity but also improves vascular function, thereby potentially contributing to the observed in vivo hemodynamic improvements.

In vivo, the polyphenol fraction produced significant improvements in both hemodynamic and renal functional parameters. The reduction in systolic blood pressure to near-normotensive levels, accompanied by substantial decreases in serum creatinine and UACR, indicates a strong protective effect against hypertension-induced renal damage. While similar effects have been reported for isolated polyphenols and pharmaceutical agents such as ACE inhibitors, the ability of a low-cost, waste-derived fraction to achieve comparable outcomes underscores its translational potential [35,36].

Mechanistically, the coordinated changes observed in NF-κB, Nrf2, and TGF-β biomarkers are consistent with modulation of inflammatory, antioxidant, and fibrotic signaling pathways. Although these findings support a plausible mechanistic framework, they do not establish direct causal regulation, and additional pathway-specific studies are required to confirm the underlying molecular mechanisms. The suppression of NF-κB p65 (2.53 to 1.19-fold) and TGF-β1 (3.75 to 1.65-fold), alongside restoration of Nrf2 expression (0.55 to 0.98-fold), suggests a rebalancing of pro-inflammatory, antioxidant, and fibrotic signaling networks [37]. This tri-axis regulation is particularly relevant in hypertensive nephropathy, where oxidative stress and inflammation drive progressive fibrosis. Previous studies have often examined these pathways in isolation [37,38]; however, the present findings highlight the importance of simultaneous modulation, which may explain the superior efficacy of the polyphenol fraction.

A notable strength of this study is the integration of gut–kidney axis analysis, which provides insight into systemic mechanisms beyond the kidney itself. Hypertension-induced dysbiosis was associated with reduced SCFAs and elevated uremic toxins, consistent with prior reports linking microbial imbalance to renal dysfunction [39,40]. The restoration of SCFAs (e.g., butyrate from 0.46 to 0.94-fold) and reduction of indoxyl sulfate (2.96 to 1.21-fold) and p-cresyl sulfate (3.05 to 1.28-fold) following treatment indicate a substantial shift toward a healthier microbial metabolic profile. The observed microbiome remodeling further supports this mechanism. The increase in beneficial taxa such as *Akkermansia* and *Lactobacillus*, alongside reductions in *Enterobacteriaceae* and *Desulfovibrio*, suggests that the polyphenol fraction promotes a more favorable microbial environment. These taxa are known to influence SCFA production and inflammatory tone [41], providing a mechanistic link between microbiome composition and metabolite profiles. The improvements in renal histology and endothelial function provide additional confirmation of the systemic effects of the polyphenol fraction. The reduction in fibrosis and IFTA scores, combined with enhanced vasorelaxation (40.0% to 75.7%), indicates that both structural and functional aspects of vascular and renal health are restored. The ability of the fraction to improve vascular responsiveness suggests potential benefits beyond renal protection, including cardiovascular risk reduction.

Despite these promising findings, several limitations should be considered. First, while the study integrates in silico, in vitro, and in vivo approaches, direct molecular validation (e.g., gene expression or protein-level mechanistic assays) was not performed. Second, the use of a single animal model may limit generalizability across different etiologies of kidney disease. Third, although the metabolomic and microbiome analyses provide strong associative evidence, causal relationships between microbial changes and renal outcomes require further investigation. Although this study integrated in silico prediction, cell-based assays, and in vivo validation to provide converging evidence for modulation of the NF-κB/Nrf2/TGF-β signaling axis, direct confirmation at the mRNA or protein expression level (e.g., RT-qPCR or Western blotting) was not performed. Likewise, the molecular docking analysis was intended to prioritize plausible target interactions and complement the experimental findings rather than establish direct ligand–target binding. Future studies employing biophysical target engagement approaches, such as surface plasmon resonance (SPR) or cellular thermal shift assay (CETSA), would provide additional mechanistic confirmation. Another limitation is the absence of comprehensive quantitative polyphenol profiling and comparative MS/MS characterization of the crude extract, although the current UHPLC–HRMS analysis was sufficient to identify the principal bioactive constituents supporting the biological findings. Therefore, the proposed molecular mechanisms should be interpreted as being supported by functional and biochemical evidence rather than definitive mechanistic validation. Future studies incorporating gene and protein expression analyses will further strengthen the mechanistic understanding of the observed nephroprotective effects. Next limitation of the present study is that the pharmacological positive control was not evaluated in the in vitro experiments. Instead, the positive control was incorporated into the in silico analyses and subsequently validated in the in vivo hypertension-associated kidney injury model, which was prioritized because it more comprehensively reflects systemic pharmacodynamic responses than isolated cellular assays. Although this design enabled direct comparison with an established therapeutic agent under physiological conditions, future studies should include positive controls in the in vitro experiments to provide a complete mechanistic comparison across all experimental levels.

In conclusion, this study provides strong evidence that a bioactivity-guided SCG polyphenol fraction exerts multi-target nephroprotective effects through integrated modulation of NF-κB/Nrf2/TGF-β signaling and the gut–kidney axis. By combining chemical enrichment with multi-scale biological validation, this work advances the understanding of how natural polyphenols can be optimized for therapeutic use. Importantly, the utilization of spent coffee grounds as a sustainable source of bioactive compounds highlights the potential of circular bioeconomy approaches in developing cost-effective interventions for chronic diseases.

## 5. Conclusions

This study demonstrates that a bioactivity-guided polyphenol-enriched fraction derived from spent coffee grounds is associated with improved renal function, reduced oxidative stress, inflammation, and fibrosis, and favorable modulation of gut–kidney axis-related metabolites in experimental models of hypertension-associated kidney injury. The observed biological effects are consistent with coordinated changes in NF-κB-, Nrf2-, and TGF-β-associated biomarkers; however, the underlying molecular mechanisms require further validation. Because the enriched fraction contains multiple phenolic constituents, the observed bioactivity is likely attributable to their combined or synergistic effects rather than to chlorogenic acid derivatives alone.

Overall, these findings provide preclinical evidence supporting the further investigation of spent coffee ground-derived polyphenol fractions as a sustainable source of nephroprotective bioactive compounds. Future studies should focus on identifying the principal active constituents, characterizing pharmacokinetic properties and safety profiles, establishing dose–response relationships, confirming molecular mechanisms through pathway-specific experiments, and evaluating efficacy in well-designed clinical studies.

## Figures and Tables

**Table 1:** In Silico Target Prediction Supports Multi-Target Modulation Across Hemodynamic and Molecular Axes.

Ligand	Target	Binding Energy (kcal/mol)	Key Interpretation
Chlorogenic acid, 5-CQA	NF-κB p65	−8.4	Hydrogen bonding with Lys218, Arg246
Chlorogenic acid, 5-CQA	TGF-beta receptor I	−8.1	Stable hinge-region interaction
Chlorogenic acid, 5-CQA	Keap1 Kelch domain	−8.7	Potential Nrf2 liberation support
Caffeic acid	NF-κB p65	−6.9	Moderate affinity
Ferulic acid	Keap1 Kelch domain	−7.1	Moderate affinity
diCQA-1	ACE	−9.0	Strong polar interaction profile
diCQA-2	AT1 receptor	−8.5	Hydrophobic pocket occupancy
Captopril (Control)	ACE	−8.0	Reference ACE inhibitor
Losartan (Control)	AT1 receptor	−8.5	Standard AT1 antagonist

**Table 2:** Targeted metabolomic profiling of gut–kidney axis-related metabolites across experimental groups.

Metabolite	Class	NC Fold	HTN Fold	HTN CAP Fold	HTN CE Fold	HTN PF-L Fold	HTN PF-H Fold
Butyrate	SCFA	1	0.46	0.86	0.69	0.77	0.94
Propionate	SCFA	1	0.58	0.88	0.73	0.79	0.96
Acetate	SCFA	1	0.71	0.93	0.84	0.89	0.98
Indoxyl sulfate	Uremic toxin	1	2.96	1.41	1.92	1.63	1.21
p-Cresyl sulfate	Uremic toxin	1	3.05	1.47	2.01	1.74	1.28
Trimethylamine N-oxide	Host-microbial co-metabolite	1	1.88	1.23	1.44	1.31	1.14
Kynurenine	Inflammation-related	1	1.76	1.19	1.35	1.28	1.09
Hippurate	Microbial co-metabolite	1	0.63	0.89	0.79	0.85	0.96

Fold values are expressed relative to the normal control group (NC = 1.0). HTN: L-NAME-induced hypertensive group; CAP: captopril-treated group; CE: crude extract-treated group; PF-L and PF-H: low- and high-dose SCG polyphenol fraction-treated groups, respectively.

## Data Availability

The original contributions presented in this study are included in the article. The datasets used and/or analysed during the current study are available from the corresponding authors on reasonable request. Further inquiries can be directed to the corresponding authors.
